# Silencing of *LINC00467* inhibits cell proliferation in testicular germ cell tumors cells

**DOI:** 10.17305/bb.2023.8969

**Published:** 2023-10-01

**Authors:** Fang Zhu, Zhizhong Liu, Qianyin Zhou, Dai Zhou, Jingyu Fan, Hao Bo, Liqing Fan

**Affiliations:** 1 Institute of Reproductive and Stem Cell Engineering, School of Basic Medical Science, Central South University, Changsha, China; 2 Department of Urology, Hunan Cancer Hospital, The Affiliated Cancer Hospital of Xiangya, School of Medicine of Central South University, Changsha, Hunan, China; 3 Clinical Research Center for Reproduction and Genetics in Hunan Province, Reproductive and Genetic Hospital of CITIC-Xiangya, Changsha, China; 4 Department of Chemistry and Biochemistry, University of South Carolina, Columbia, SC, United States

**Keywords:** Testicular germ cell tumors (TGCTs), *LINC0046*, dihydrotestosterone (DHT), cell proliferation, cell cycle

## Abstract

A significant decrease in *LINC00467* expression in testicular germ cell tumors (TGCTs) was found in our previous study in comparison to adjacent tissue. The expression of *LINC00467* correlated with the pathological grade of the tumor in TGCT patients. The higher the expression of *LINC00467* was, the worse the prognosis of the patients with TGCT was. Despite these findings, the exact role of *LINC00467* in the development of TGCTs requires further investigation. *LINC00467* expression was downregulated in the NCCIT and TCam-2 cell lines via small interfering RNA (siRNA) silencing. The levels of gene expression were validated using quantitative real-time polymerase chain reaction (qRT-PCR) analyses. Cell proliferation was evaluated by the MTT and Cell Counting Kit-8 (CCK8) assays, whereas flow cytometry was used to assess the effects on the cell cycle. Western blotting analysis was used to detect expression levels of protein. Additionally, RNA-sequencing and bioinformatics methods were used to investigate the mechanism of action of *LINC00467* in TGCTs. The suppression of *LINC00467* expression resulted in decreased cell proliferation and induced S-phase arrest. Furthermore, the suppression of *LINC00467* downregulated proliferating cell nuclear antigen (PCNA), a protein related to cell cycle regulation, while it upregulated p21 expression. In other studies involving dihydrotestosterone (DHT) stimulation, it was observed that DHT could upregulate *LINC00467* expression. In addition, silencing of the *LINC00467* reversed the effect of testosterone on cell proliferation. The Gene Set Enrichment Analysis (GSEA) revealed that *LINC00467* regulated the p53 pathway by modulating the expression of *CCNG1*. Our study found that *LINC00467* regulates cell proliferation by inducing S-phase arrest through the cell cycle-related proteins PCNA and p21. These findings contribute to our understanding of non-coding RNAs mechanisms involved in the development of TGCTs.

## Introduction

Testicular cancer occurs frequently, particularly among young men aged 15–44 years old [1, 2]. Approximately 98% of testicular cancers are marked as testicular germ cell tumors (TGCTs), which are solid tumors originating from the primitive germ cells of the testis. The main types of TGCTs are seminoma and non-seminoma, with seminoma originating from the primordial germ cells of the testis, and non-seminomatous tumor cells exhibiting embryonic or extra-embryonic differentiation, including embryonal carcinoma, yolk sac tumor, and choriocarcinoma [3]. Orchiectomy combined with radiotherapy is a common treatment method for most patients and it yields positive outcomes [4, 5]. However, the underlying pathogenesis of TGCTs is still not well understood, leaving patients susceptible to a range of problems after treatment [6]. Such problems include possible side effects of radiotherapy and an increased likelihood of developing a second form of malignancy, such as secondary non-germ cell cancers, as well as cardiovascular disease, or hypogonadism [7–9]. Therefore, more comprehensive knowledge of the TGCTs’ pathogenesis is necessary to develop more effective treatment methods and diagnostics.

Long-stranded non-coding RNAs (lncRNAs) are RNA molecules that rarely code proteins and they are longer than 200 nucleotides. They can be divided into several types, including sense, antisense, bidirectional, intragenic, and intergenic lncRNAs. Recent research has identified the majority of transcribed sequences in the human genome as lncRNAs [10]. These RNA molecules can regulate gene expression through various mechanisms, such as transcriptional regulation, transcription factor trapping, chromatin circularization, and gene methylation [11]. Dysregulation of lncRNA expression has been linked to several diseases including cancer [12]. For instance, studies have suggested that lncRNA *MALAT1* may promote tumor growth and metastasis and could be a potential prognostic marker for oral squamous cell carcinoma and non-small cell lung cancer [13, 14]. However, the research on the role of lncRNAs in the development of TGCTs is limited.

**Table 1:** Primer sequences used in the process of RNA extraction

**Primer**	**Forward primers**	**Reverse primers**
*LINC00467*	TCGTCTTCAGGAAGCCAGAC	TGGAAATCAAAAGGGTCAGC
*CCNG1*	TGCTGAAGCTGAGGACACAC	CCCAGATGCTTCCGTTCTTA
*HAVCR2*	TGTGCCTAACAGAGGTGTCC	CGACCTCCGCTCTGTATTCC
*TIGIT*	GCCTAGGGTGAGTAACGTGG	CTGCAGTCATGCCATCCTCA
Beta actin	GTGGCCGAGGACTTTGATTG	CCTGTAACAACGCATCTCATATT

*LINC00467*, also known as *C1orf97*, is a lncRNA located on chromosome 1, with a full length of 2028 base pairs. This lncRNA is highly expressed in normal testis but has lower or no expression in other tissues [15]. The mechanism of regulation of the *LINC00467* expression still remains unknown. Research has shown that *LINC00467* plays a significant role in the development of various cancers. It was first discovered that *LINC00467* promotes the survival of neuroblastoma cells and inhibits apoptosis by cis-regulating the expression of its neighboring gene, *RD3* [16]. *LINC00467* also targets microRNA *451a*, which promotes carcinogenesis and metastasis in human colorectal cancer [17]. In hepatocellular carcinoma, the LINC00467/miR-9-5p/PPARA pathway plays a critical role in cancer occurrence and progression [18].

Our previous study has shown a significant decrease in *LINC00467* expression in TGCTs, compared to adjacent tissue. Furthermore, it was found that *LINC00467* expression was upregulated in TGCTs at stages II/III in contrast to the stage I of TGCT. Additionally, a negative correlation between LINC00467 expression and both the 5-year overall survival and the 5-year disease-free survival was observed [15]. This implicates that although *LINC00467* is downregulated in TGCT, it may play an oncogene-like function in TGCT. Also, we have found that *LINC00467* has a testicle-specific expression of lncRNA, and this expression pattern is very similar to the pattern of testicular tumor antigen, which is highly likely to have important functions. Therefore, *LINC00467* has important application prospects as a tumor therapeutic target or biomarker. We conducted an in-depth study by knocking down *LINC00467* in TGCT cell lines to determine its specific function and molecular mechanism in the TGCTs. The results of our study may provide new insight into the diagnosis and targeted therapy of TGCTs and could also contribute to the development of diagnostic markers and therapeutic targets for other cancers.

## Materials and methods

### Cell culture and siRNA transfection

The TCam-2 seminoma cell line was generously donated by Prof. Tang Yuxin [19], while the non-seminoma human embryonic carcinoma cell line NCCIT was purchased from the American Type Culture Collection (ATCC) based in Virginia, USA. TCam-2 and NCCIT cells were cultured in Dulbecco’s Modified Eagle’s Medium (DMEM) and RPMI-1640 medium, respectively (both from GIBCO, USA). All cells were cultured in a medium containing 10% fetal bovine serum (FBS; GIBCO, USA), 100 U/mL penicillin, and 100 µg/mL streptomycin (GIBCO, USA), in a 5% CO_2_ incubator at 37 ^∘^C. Transfection was performed when the cells had grown to 60%–80% confluence, and the lipofectamine 3000 transfection kit (cat no. L3000015, ThermoFisher) was utilized strictly following provided instructions. The *LINC00467* siRNA sequences used were *LINC00467*-siRNA-1: GTCTTCAGGAAGCCAGACA and *LINC00467*-siRNA-2:GATGCTCTGTAAACCACAT. Both the nucleotide sequences of the non-target negative control (NC) and *LINC00467* siRNA sequences were synthesized by RiboBio (Guangzhou, China).

### RNA extraction and qRT-PCR

The TRIzol method was used to extract RNA (cat no. 15596018, ThermoFisher). A 1–2 µL of RNA was used to determine its concentration and quality using a spectrophotometer, and another 1–2 µL of RNA was used for gel electrophoresis to determine the quality of the RNA. A Roche reverse transcription kit (Transcriptor First Strand cDNA Synthesis Kit, cat no. 4897030001, Roche) was used for the reverse transcription PCR reaction. Levels of target mRNAs were measured using qRT-PCR (LightCycler 480 SYBR Green I Master, 10x, cat no. roche04887352001, Roche) after transfection for 48 h, and β-actin was used for normalization. The primer sequences are shown in Table 1.

### MTT assay

The cells were seeded into 96-well plates at a concentration of 2000 cells per well in 3 replicate wells after transfection 48 h. At 24, 48, 72, 96, and 120 h, 20 µL of MTT solution (5 mg/mL) was added to each well. After incubation in a 5% CO_2_ atmosphere at 37 ^∘^C for 4 h, the supernatant in each well was discarded. Then, 150 µL of dimethylsulfoxide (DMSO) was added to each well, and placed on a shaker for 1 min. The absorbance of each well was measured at 492 nm using a microplate reader. The absorbance value was used as the ordinate and the interval time was used as the abscissa, while the MTT cell growth curve was drawn using GraphPad Prism 5.0 software.

### Cell Counting Kit 8 (CCK8) assay

The cells were seeded into 96-well plates at a concentration of 2000 cells per well in 3 replicate wells after transfection 48 h. At 24, 48, 72, and 96 h, 10 µL of CCK-8 solution (Sangon Biotech) was added to each well. After incubation in a 5% CO_2_ atmosphere at 37 ^∘^C for 2 h, the mixture was shaken for 1 min on a shaker in the dark. Then, absorbance at 450 nm was measured using a microplate reader. The absorbance value was used as the ordinate and the interval time was used as the abscissa. The CCK8 cell growth curve was drawn using GraphPad Prism 5.0 software.

### Cell cycle assays

The cells were mixed with 95% ice-cold ethanol and fixed at 4 ^∘^C for 16 h after transfection 48 h. Then, the cells were centrifuged at 300 *g* for 3 min and the supernatant was discarded. The cells were washed by resuspending them in ice-cold PBS. Then, 0.4 mL of propidium iodide staining solution (Cell Cycle and Apoptosis Analysis Kit, cat no. FXP021-200, Beijing 4A Biotech Co., Ltd.) was added to each tube of cells slowly and the cell was fully resuspended and incubated at 37 ^∘^C for 30 min in the dark. The red fluorescence was detected at an excitation wavelength of 535 nm using a BD flow cytometry analyzer. The light scattering pattern was detected at the same time. Cell cycle analysis was performed using Modfit software.

### Cell apoptosis assay

The cells were resuspended in a 1×Annexin-binding buffer and the cell concentration was adjusted to 1×10^6^ cells/mL. Then, 100 µL of the cell suspension from each sample was taken and 5 µL of Alexa Fluor^®^ 488annexin V and 1 µL of PI (100 µg/mL) (FITC Annexin V Apoptosis Detection Kit I, cat no. 556547, BD) was added. The mixture was kept to incubate at room temperature for 15 min in the dark. Then, 400 µL of 1×annexin-binding buffer was added to each sample and mixed by vortexing, and then a BD C6 flow cytometer was used for detection. Cell apoptosis analysis was performed using Flowjo 10 software.

### Dihydrotestosterone (DHT)-treated cells

The cells were seeded into 6-well plates at an appropriate density. After the cells had reached 70% confluence overnight, the cells were washed twice with 1×PBS, and complete culture mediums containing 0 nM/mL, 1 nM/mL, and 5 nM/mL DHT (cat no. S4757, Selleck) were prepared using the complete medium as previously described. Then, 2 mL of complete medium was added at the above concentrations to each well on the six-well plate using three wells for each concentration. The cells were cultured in a 5% CO_2_ atmosphere at 37 ^∘^C for 36 h and then the cells were collected.

### Western blotting analysis

The protein precipitates were extracted using an RIPA protein lysate (cat no. 89900, Thermo Fisher) containing a protease inhibitor phenylmethylsulfonyl fluoride (cat no. 36978, Thermo Fisher, USA). Protein concentrations were determined using a BCA kit (ThermoFisher, USA). Then, the protein was subjected to SDS polyacrylamide electrophoresis and transferred onto a PVDF membrane (cat no. IPVH00010, Millipore). The PVDF membrane was blocked with 5% skimmed milk at room temperature. Then, the PVDF membranes were incubated overnight at 4 ^∘^C with primary antibodies diluted with 5% skimmed milk. The used primary antibodies were mouse anti-PCNA monoclonal antibody (1:2000, cat no. ab29, Abcam, USA) and rabbit anti-p21 polyclonal antibody (1:1000, cat no. 10355-1-AP, Proteintech, Chian). Rabbit anti-vinculin monoclonal antibody (1:5000, cat no. ab129002, Abcam, USA) was used as the loading control. Then, the appropriate secondary antibody (CoWin Biosciences) was incubated with the PVDF membranes based on the genetic origin of the primary antibody. A gel imaging system was used to scan the protein bands using ECL reagents, and vinculin was used as an internal reference.

### RNA-sequencing

The RNA-sequencing was performed as previously described [20] There were four samples of TCam-2 that have been sequenced, two from each group (NC vs siRNA). RNA sequencing was performed through de-ribosomal strand-specific library sequencing by first removing rRNA from total RNA to retain the mRNAs and ncRNAs. The enriched mRNAs and ncRNAs were fragmented into short fragments. The cDNA fragments were purified using a QiaQuick PCR extraction kit to repair the ends, add poly(A), and ligate the Illumina sequencing joints. The second-strand cDNA was digested with uracil-N-glucanase (UNG). Agarose gel electrophoresis was conducted to determine the size of the digested products. Then, the products were obtained and amplified using PCR and sequenced using an Illumina HiSeqTM 4000 platform (Gene Denovo, Guangzhou, China). The resulting cDNA library was sequenced using an Illumina Novaseq6000 system (Gene Denovo Biotechnology Co., Guangzhou, China).

### Gene set enrichment analyses (GSEA)

GSEA determines whether a pre-defined set of genes shows statistically significant differences in the gene expression profiling of the NC group vs the *LINC00467* silencing group. An annotated gene set (https://www.gseamsigdb.org/gsea/msigdb/genesets.jsp?collection=CP:KEGG) was selected as the reference gene set. The Hiplot (https://hiplot.com.cn/) was used for the enrichment analysis at default parameters.

### Ethical statement

Human samples and animals were not involved in this study.

### Statistical analysis

The Student’s *t*-test was used to determine the significance of differences between the two groups. GraphPad Prism Software 8.0 was used to analyze the statistics. A *P* value < 0.05 was considered to indicate a significant difference.

## Results

### Silencing of *LINC00467* inhibited the proliferation of TGCT cells

**Figure 1.**
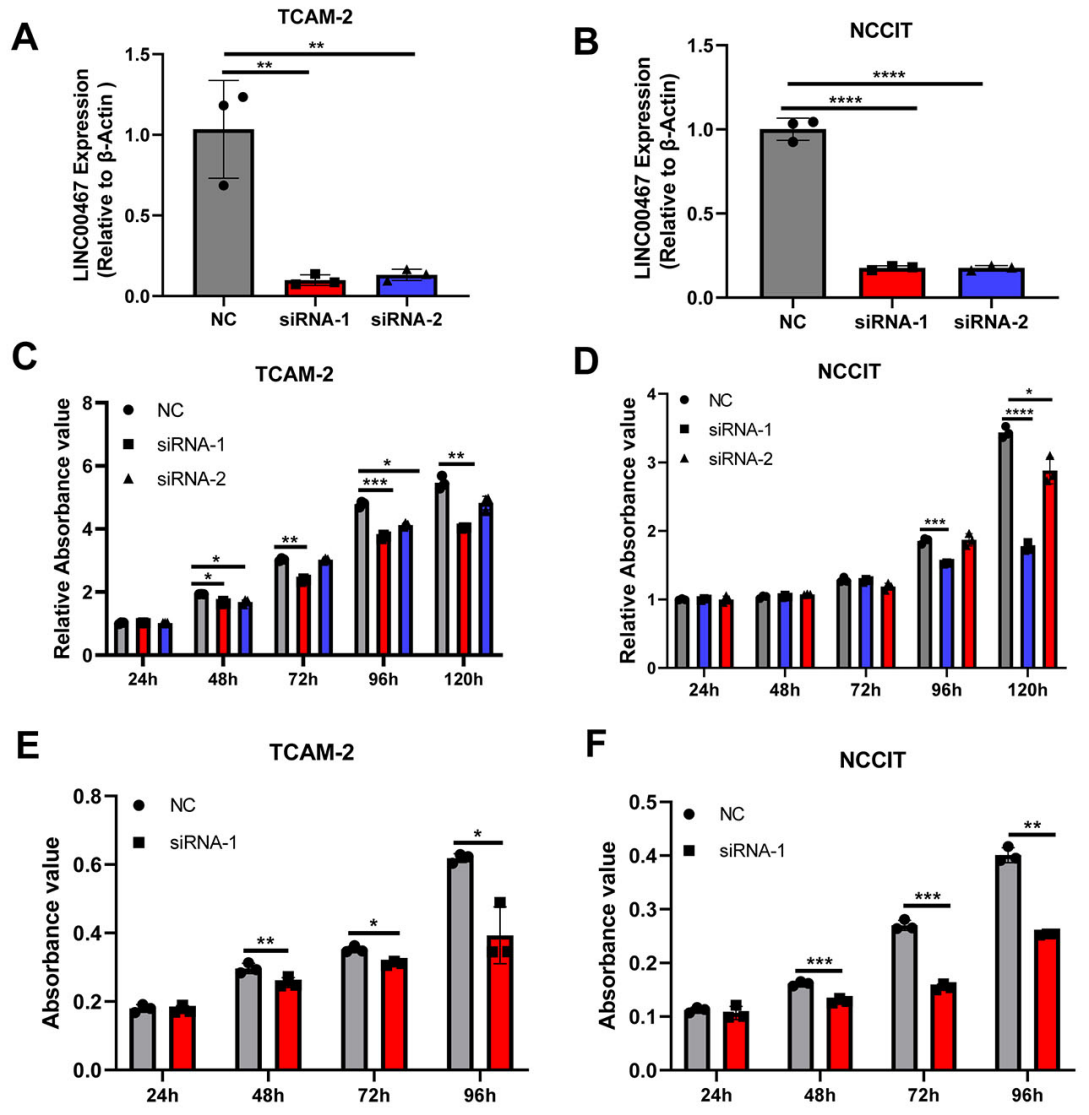
**Silencing of *LINC00467* inhibited TGCT cell proliferation.** Silencing efficiency of *LINC00467* in TCam-2 (A) and NCCIT (B) cell lines; (C and D) The proliferation of cells in the NC group and *LINC00467-*silenced group (siRNA) was detected using an MTT assay; (E and F) The proliferation of cells in the NC group and *LINC00467*-silenced group was detected using CCK8 assay. *P value < 0.05; **P value < 0.01; ***P value < 0.001; ****P value < 0.0001. TGCT: Testicular germ cell tumor; siRNA: Small interfering RNA; NC: Negative control; CCK8: Cell Counting Kit 8.

We used siRNA to silence the expression of *LINC00467* in TCam-2 and NCCIT cells and detected the silencing efficiency using qRT-PCR (Figure 1A and 1B). The cell proliferation of TGCT cells was analyzed using CCK8 (Figure 1C and 1D) and MTT (Figure 1E and 1F) assays. *LINC00467* silencing inhibited cell proliferation, compared with the NC group (Figure 1C–1F).

### Silencing of *LINC00467* induced S-phase arrest but did not affect apoptosis

**Figure 2.**
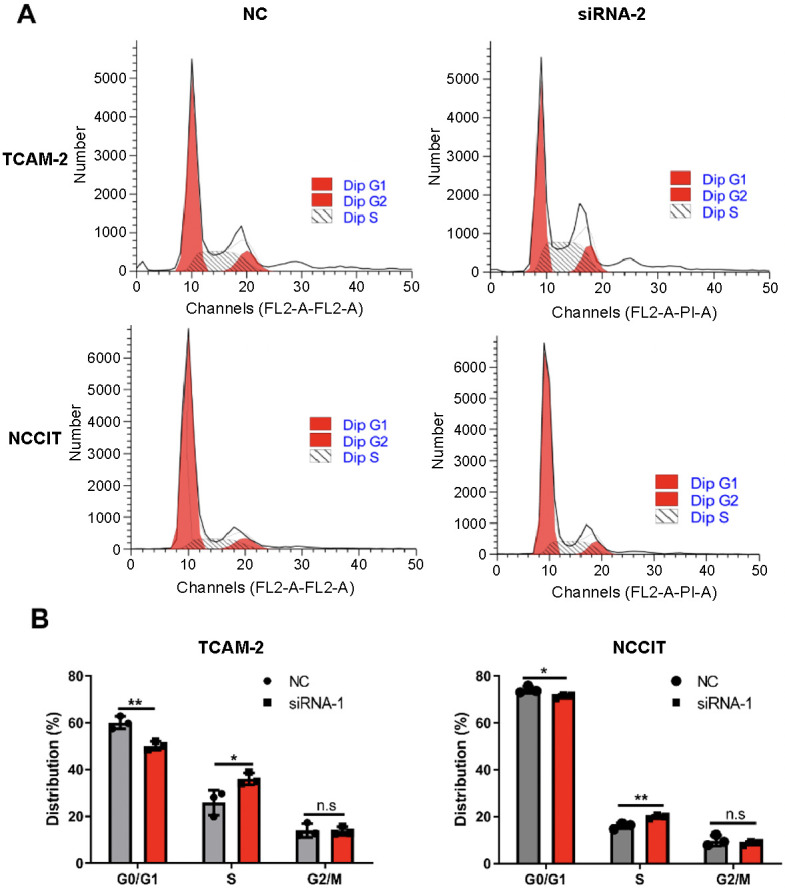
**Silencing of *LINC00467* inhibited TGCT cell cycle progression.** (A) Determination of cell cycle distribution after *LINC00467* silencing using flow cytometry. Cell cycle analysis of TCam-2 (B) and NCCIT (C). Error bars indicate SD (*n* ═ 3). *P value < 0.05; **P value < 0.01. TGCT: Testicular germ cell tumor; siRNA: Small interfering RNA; NC: Negative control.

**Figure S1.**
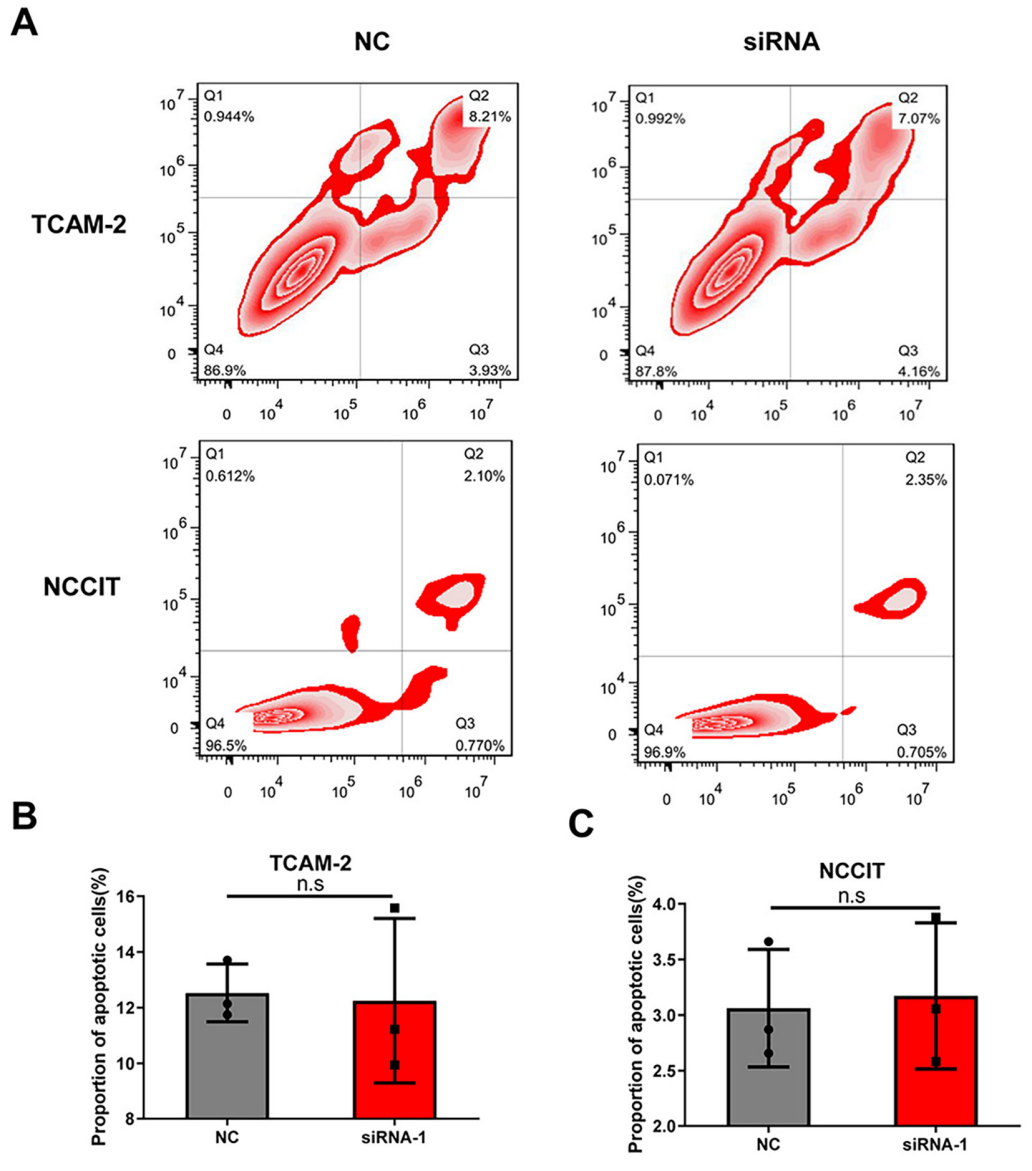
**Silencing of *LINC00467* did not affect the apoptosis of TGCT cells.** (A) Apoptosis was determined using flow cytometry analysis of Annexin V/PI double-stained TCam-2 and NCCIT cell lines. Apoptosis rate in the two cell lines: siRNA group (B) vs NC group (C). n.s: Not statistically significant; TGCT: Testicular germ cell tumor; siRNA: Small interfering RNA; NC: Negative control.

Flow cytometry assays were used to examine the effect of *LINC00467* silencing on the cell cycle (Figure 2A). It showed that *LINC00467* silencing affected cell cycle distribution and induced S-phase arrest (Figure 2B and 2C), compared with the NC group. Next, to examine the effect of *LINC00467* silencing on cell apoptosis, Annexin V/PI double staining was performed, followed by flow cytometry analysis (Figure S1A). *LINC00467* silencing had no effect on cell apoptosis, compared with the NC group (Figure S1B and S1C). Together, these results indicate that *LINC00467* may exert a cancer-promoting effect.

### Silencing of *LINC00467*-regulated cell cycle-related proteins

**Figure 3.**
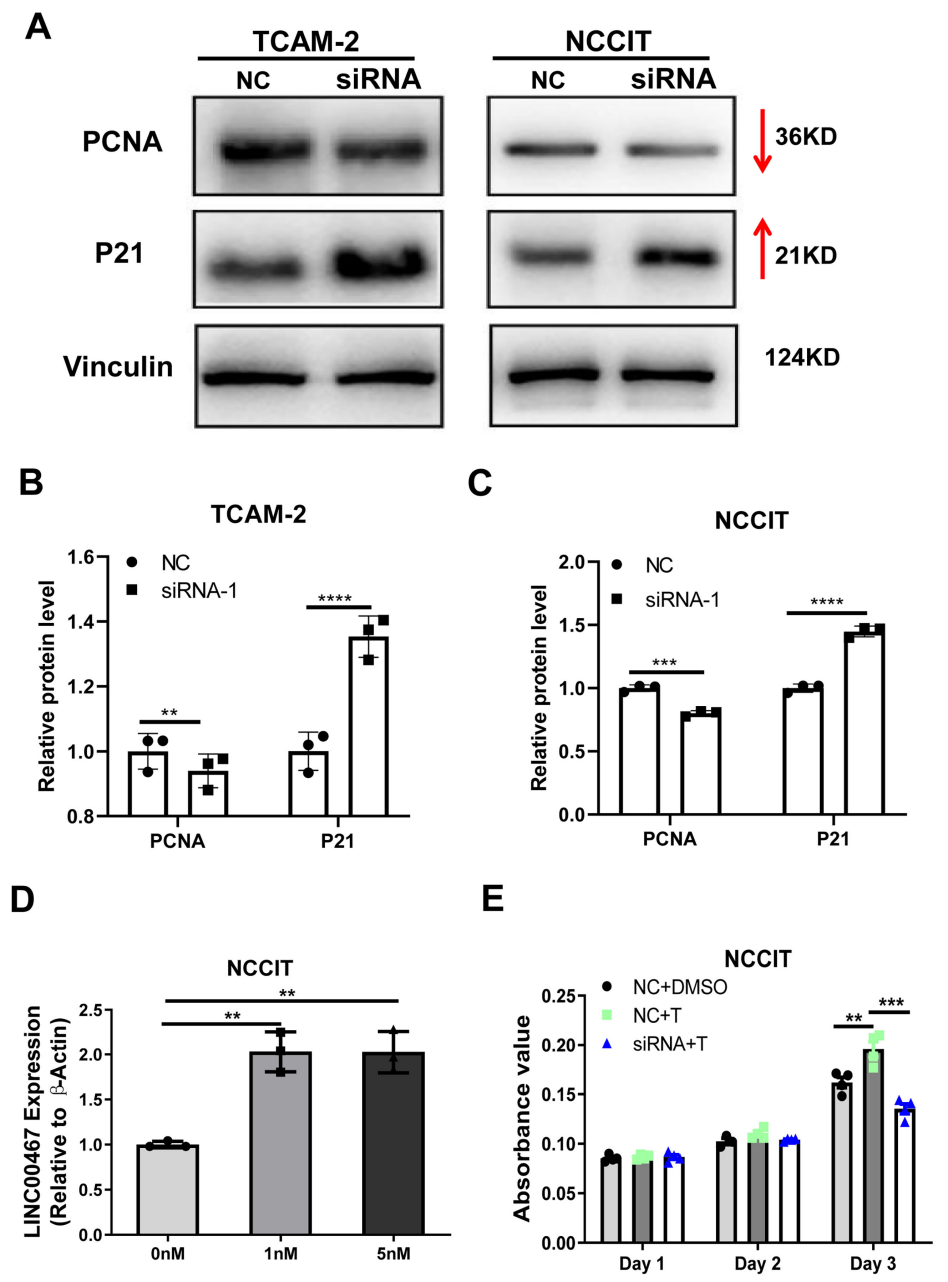
**LINC00467 silencing regulated cell cycle-related protein expression and inhibited the effect of DHT on cell proliferation.** (A) Western blotting analysis of the change in the p21 and PCNA protein level after *LINC00467* silencing. The relative expression of p21 and PCNA in each group was calculated using vinculin expression as an internal reference and normalized to one in the NC group of TCam-2 (B) and NCCIT (C); (D) DHT increased the expression of *LINC00467*; (E) The silencing of *LINC00467* inhibited the effect of DHT on cell proliferation. **P value < 0.01; ***P value < 0.001; ****P value < 0.0001. DHT: Dihydrotestosterone; PCNA: Proliferating cell nuclear antigen; siRNA: Small interfering RNA; NC: Negative control; DMSO: Dimethylsulfoxide.

After identifying the function of *LINC00467*, we attempted to investigate its molecular mechanism. Based on the aforementioned cell proliferation detection and cell cycle detection results, after silencing *LINC00467* in TCam-2 and NCCIT cells, we detected key node molecules, such as p21 and PCNA, in the cell cycle signaling pathway (Figure 3A). We found that the silencing *LINC00467* resulted in a decrease in PNCA protein expression in the two cell lines, as well as the upregulation of the p21 protein expression (Figure 3B and 3C).

### Silencing of *LINC00467* reversed the effect of testosterone on cell proliferation

Androgens have a robust impact on cell proliferation. Therefore, we aimed to verify whether testosterone exerts a regulatory effect on *LINC00467*, which affects cell proliferation. First, we tested the effect of different concentrations of DHT (0–5 nM/mL) on the expression of *LINC00467* in cultured NCCIT cell lines using qRT-PCR. Interestingly, both 1 nM/mL DHT and 5 nM/mL DHT upregulated the expression of *LINC00467* (Figure 3D). Further, MTT experiments showed that silencing *LINC00467* could significantly inhibit the promotional effect of DHT on cell proliferation (Figure 3E). Our results showed that *LINC00467* is required for the testosterone to promote the cell proliferation.

### Gene set enrichment analyses (GSEA) showed that *LINC00467* silencing was related to the p53 signaling pathway

**Figure 4.**
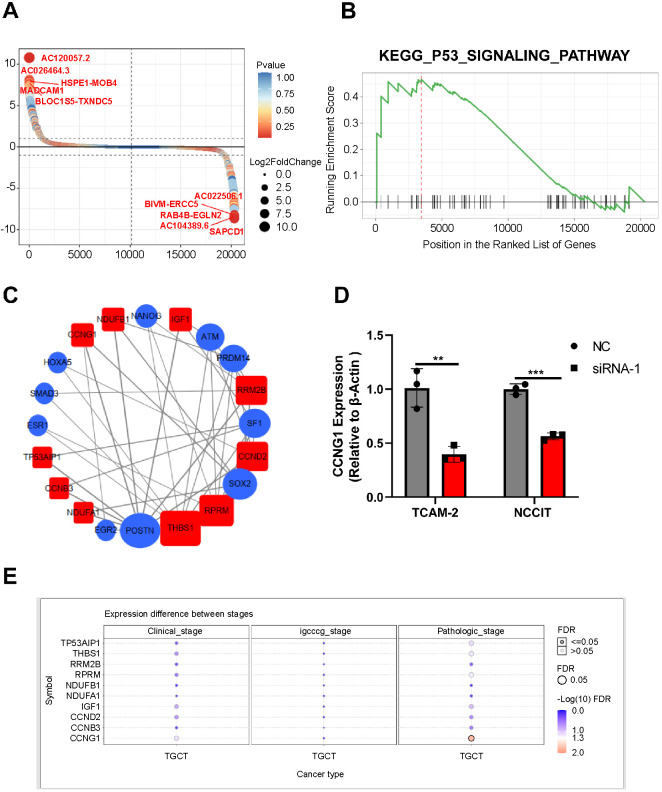
**Identification of *LINC00467*-related signaling pathways using GSEA.** (A) Gene differential ranking diagram showing the hierarchical clustering of mRNAs in the NC group and siRNA group; (B) GSEA showed that mRNA expression was enriched in the p53 signaling pathway; (C) The RNA network of the genes of the p53 signaling pathway and their transcription factor in the NC group vs siRNA group. Red color: The genes of the p53 signaling pathway in the NC group vs siRNA group. Blue color: The transcription factor of the “red” gene. (D) The expression of *CCNG1* in the NC group and siRNA group was detected using qRT-PCR; (E) Correlation between the p53 pathway genes and TGCT grade distribution in the NC group vs siRNA group. **P value<0.01; ***P value < 0.001. GSEA: Gene set enrichment analysis; NC: Negative control; siRNA: Small interfering RNA; qRT-PCR: Quantitative real-time polymerase chain reaction; TGCT: Testicular germ cell tumor.

To further explore the specific effects of *LINC00467* on the proliferation of TGCT cells, we used RNA-sequencing to detect the expression profile of TCam-2 cells after silencing *LINC00467*. The expression levels of many genes were found to be regulated by the silencing *LINC00467* (Figure 4A). Based on RNA-sequencing data, we explored the *LINC00467-*related signal pathway using GSEA. The p53 signaling pathway was enriched in the gene expression dataset following the silencing of *LINC00467* (Figure 4B). The network of the p53 signaling pathway genes and their transcription factors based on our RNA-seq data revealed that *LINC00467* may be able to regulate the p53 signaling pathway through multiple transcription factors, including the transcription factors, NONAG, and HOXA5 (Figure 4C). One of the p53 signaling pathway genes, *CCNG1*, was associated with the distribution of TGCT pathological grades (Figure 4E). The expression of *CCNG1* was also significantly downregulated in the *LINC0046-*silencing group, which was validated by qRT-PCR (Figure 4D). These results indicate that the silencing of *LINC00467* is associated with the p53 signaling pathway and that this pathway may be involved in the progression of TGCT.

### Silencing of *LINC00467* upregulated the expression of immune checkpoints

**Figure 5.**
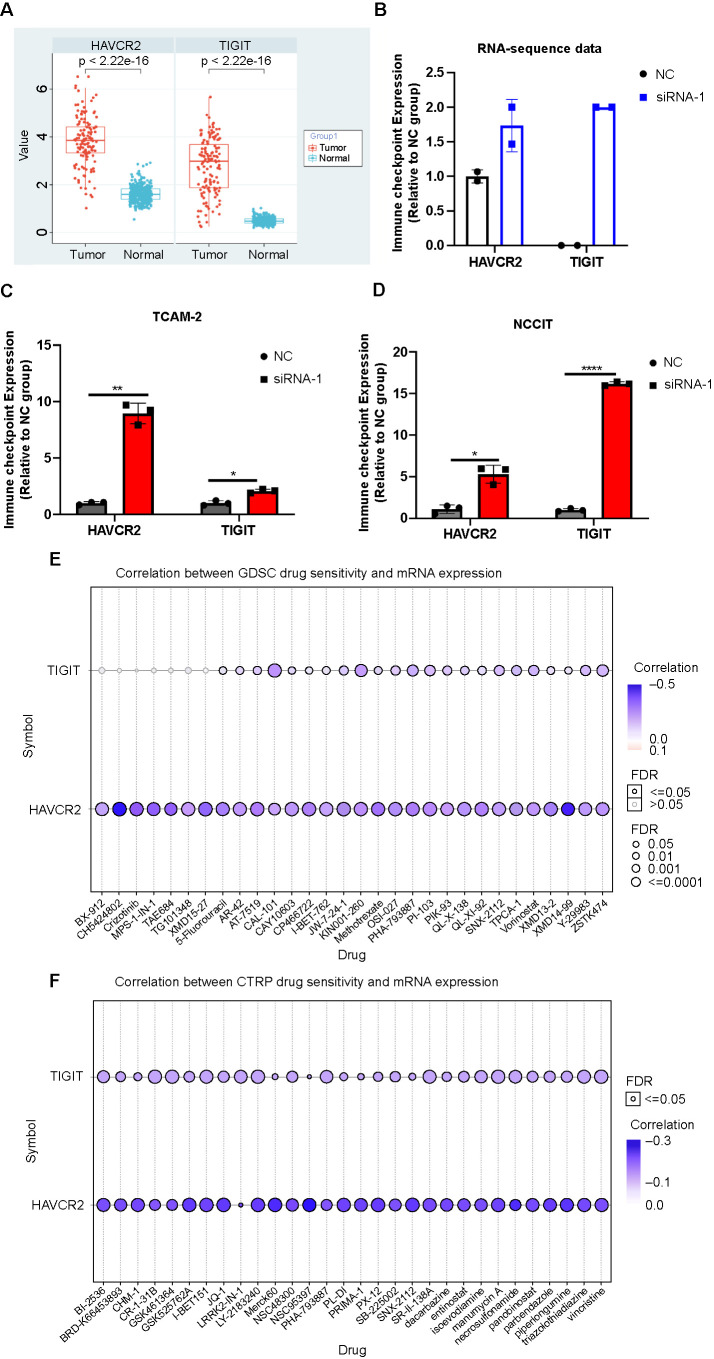
**Silencing of *LINC00467* upregulated the expression of the *HAVCR2* and *TIGIT* immune checkpoints.** (A) Expression of the immune checkpoints, *HAVCR2* and *TIGIT*, in TGCT and normal tissues; (B) *HAVCR2* and *TIGIT* expression increased in the *LINC00467*-silenced group. The expression of *HAVCR2* and *TIGIT* in TCam-2 (C) and NCCIT (D). *P value < 0.05; **P value < 0.01; ****P value < 0.0001. (E) Correlation between GDSC drug sensitivity and the expression of the *HAVCR2* and *TIGIT* immune checkpoints. (F) Correlation between CTRP drug sensitivity and the expression of the *HAVCR2* and *TIGIT* immune checkpoints. TGCT: Testicular germ cell tumor; GDSC: Genomics of Drug Sensitivity in Cancer; CTRP: Cancer Therapeutics Response Portal; NC: Negative control; siRNA: Small interfering RNA.

**Figure S2.**
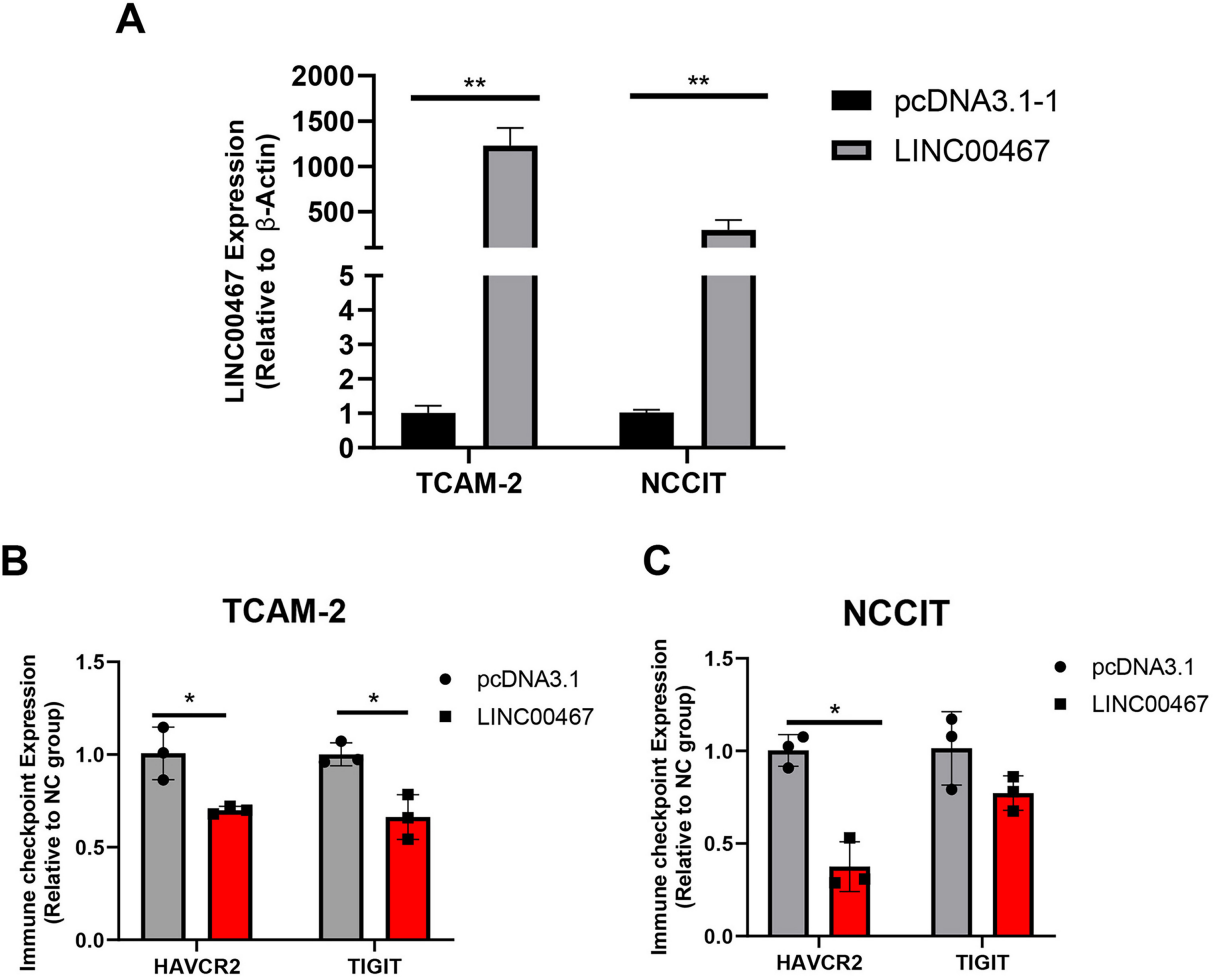
***LINC00467* downregulated the expression of the *HAVCR2* and *TIGIT* immune checkpoints.** (A) qRT-PCR assay was used to detect *LINC00467* overexpression in the cell lines. The expression of *HAVCR2* and *TIGIT* in TCam-2 (B) and NCCIT (C). *P value < 0.05. qRT-PCR: Quantitative real-time polymerase chain reaction.

Immune checkpoints are very important for the development of tumor immunotherapy and immunotherapy drugs. Two immune checkpoints, *HAVRC2* and *TIGIT*, were found to be significantly upregulated in TGCT compared with adjacent tissue by analyzing The Cancer Genome Atlas (TCGA) databases (Figure 5A). After analyzing our RNA-sequencing data, it was found that *HAVRC2* and *TIGIT* were differentially upregulated after the silencing of *LINC00467* (Figure 5B). In addition, the expression of *HAVRC2* and *TIGIT* were upregulated by the silencing of *LINC00467*, as shown through qRT-RNA validation (Figure 5C and 5D). The expressions of *HAVRC2* and *TIGIT* were downregulated by upregulating *LINC00467* (Figure S2). Furthermore, the negative correlation between Genomics of Drug Sensitivity in Cancer (GDSC) (CH542802 and XMD14-99) with *HAVCR2* expression indicated that the higher the expression of *LINC00467* was, the less sensitive the CH542802 and XMD14-99 drugs were (Figure 5E). The negative correlation between Cancer Therapeutics Response Portal (CTRP) drug sensitivity and the expression of *HAVCR2* and *TIGIT* also showed that the higher the expression of *LINC00467* was, the less sensitive CTRP drugs were, such as I-BET151, SR-II-138A, and triazolothiadiazine (Figure 5F). The above results are significant for the selection of targeted drugs for the clinical treatment of TGCTs.

## Discussion

TGCTs are malignant tumors with important genetic component in their development. Therefore, they tend to cluster within families [21]. There are approximately 50 susceptibility genes identified that contribute to the polygenic genetic risk of TGCTs [22]. However, the functional role of these gene products in the development of TGCTs is still unclear, and the underlying molecular mechanisms are complex. Recent studies show that *SPRY4-IT1* acts as an oncogene in the TGCT development, while *miR-302/367* and *miR-371-373* clusters are highly expressed in tumor tissues and serum samples of TGCT patients [23–27]. Additionally, *AKAP4* and *TNP1* are associated with TGCT development by regulating immune cell infiltration in the TGCT immune microenvironment [28]. Furthermore, *AXIN1* has been found to reduce PI3K/AKT/mTOR signaling and act as a tumor suppressor in TGCT cells [29]. These results suggest that both genetic and epigenetic factors play a role in TGCT development, and that lncRNAs may act as either oncogenes or tumor suppressors in TGCTs.

We have found that *LINC00467* is a testicle-specific lncRNA. This expression pattern is very similar to that of testicular tumor antigen and is likely to have important functions. Therefore, it has important application prospects as a tumor therapeutic target or biomarker. Our previous research has shown that *LINC00467* can activate the AKT signaling pathway by binding to *AZGP1*, leading to its degradation and promoting the progression of non-small cell lung cancer [30]. Additionally, another study by our team demonstrated that *LINC00467* is influenced by copy number amplification and DNA demethylation, making it a potential biomarker for breast cancer diagnosis and prognosis [31]. In our current study, we found that inhibiting *LINC00467* expression can suppress the proliferation of TGCTs cells. It is likely that *LINC00467* is involved in the development of TGCT by promoting the proliferation of tumor cells.

Furthermore, we discovered that silencing *LINC00467* triggered S-phase cell cycle arrest without affecting apoptosis. To further understand how *LINC00467* performs its function, we measured two proteins involved in the cell cycle, PCNA and p21. PCNA is a nuclear cofactor for DNA polymerase delta and a marker protein for cell proliferation and DNA replication. It can enhance the processability of DNA polymerase during leading strand elongation and it induces strong stimulation of 3’–5’ exonuclease and 3’-phosphodiesterase, which enhance the processivity of the leading strand during DNA replication [32]. PCNA also plays a critical role in DNA damage response by positioning itself at replication forks to coordinate DNA replication [32]. Our western blot results showed that PCNA expression decreased significantly following *LINC00467* silencing, indicating that *LINC00467* may play a role in DNA synthesis and other stages of cell proliferation.

The p21 protein binds to and inhibits the phosphorylation activity of CDK2 or CDK4 complexes, arresting cell cycle progression in the G1 phase. Expression of this protein is closely regulated by the tumor suppressor protein, p53, which responds to various stress stimuli, including DNA damage, by mediating p53-dependent G1 arrest of the cell cycle. The p21 also inhibits DNA synthesis by competing with *POLD3* for PCNA binding. Overall, *LINC00467* plays a regulatory role in DNA replication and DNA damage repair. Our experimental results showed that the expression of p21 was significantly upregulated after the silencing of *LINC00467*. Interestingly, we found that the cell cycle was not arrested at the G1 phase, but at the S-phase. This suggests that *LINC00467* may act through the unconventional p21-dependent cell cycle regulation mode, and its specific mode of action remains to be further elucidated [33]. Our experiments have demonstrated that upon the silencing of *LINC00467*, the expression of p21 was considerably increased. It is worth noting that we observed the arrest of the cell cycle not during the G1 phase, but rather at the S-phase. In the study of the mechanism of *LINC00467*, we have preliminarily confirmed that *LINC00467* can promote the process of the cell cycle by regulating the expression levels of PCNA and p21. Thus, it plays a role in regulating the occurrence of TGCT. However, the finer molecular mechanisms involved, such as how *LINC00467* regulates cell cycle-related proteins and whether *LINC00467* can also participate in the regulation of gene expression through cis-regulation as it does in other tumors, are still worth our further in-depth research and discussion.

Numerous studies have reported that an increase in testosterone leads to the proliferation of epithelial cells and adipocytes [34, 35]. In our study, cell experiments were conducted to confirm that adding DHT to cells stimulates the expression of *LINC00467*. Subsequently, we found that the silencing of *LINC00467* significantly inhibits the promotional effect of DHT on cell proliferation. Our findings suggest that *LINC00467* could be a crucial component in regulating DHT-induced cell proliferation, which provides a new perspective for understanding the development of TGCTs.

To further explore how *LINC00467* affects TGCT cell proliferation, RNA-sequencing was performed on *LINC0046* -silenced TCam-2 cells. The enrichments analysis indicated that the p53 signaling pathway was the crucial biological signaling pathway enriched in the *LINC00467*-silenced group. One of the genes involved in the p53 signaling pathway, *CCNG1*, is also associated with the distribution of TGCT pathological grades. It is suggested that *LINC00467* regulates the downstream p53 signaling pathway by regulating the expression of *CCNG1*. Moreover, our previous research found that *LINC00467* played a vital role in the invasion and metastasis of TGCT, which implies that *LINC00467* regulates the invasion and metastasis of TGCTs while cell proliferation is a contributor to TGCTs development.

Immune checkpoints play monumental roles in regulating the immune system and retain key potentials in hindering the development and autoimmunity of malignancies [36]. At present, immune checkpoint therapy is used as a cancer therapy in addition to radiation, chemotherapy, and surgery and includes the blocking of cytotoxic T-lymphocyte-associated protein 4 (CTLA-4) and programmed cell death protein 1 (PD-1) [37]. Our previous study suggests that *LNC00467* may exert an inhibitory effect on the infiltration and activation of immune cells and may be a predictive target for anti-PD-1 immunotherapy [15, 31]. To further discuss the clinical significance of *LINC0046*, we analyzed the role of immune checkpoint in *LINC00467* immunotherapy. To explore whether *LINC00467* regulates the expression of immune checkpoints, we analyzed our RNA-sequencing data and performed qRT-PCR validation. The results showed that *LINC00467* silencing leads to increased expression of *HAVRC2* and *TIGIT*. Both immune checkpoints were found to be significantly upregulated in TGCT, as shown by the TCGA data analysis. According to our previous study, the higher the expression of *LINC00467* was, the lower the rate of TGCT patient overall survival and progression-free survival was [15]. These results indicate that *LINC00467* directly regulates the expression of *HAVRC2* and *TIGIT*, which can provide novel insights for developing new treatment strategies for TGCT.

A series of recent studies have provided evidence that circulating nucleic acids, specifically extracellular nucleic acids, found in body fluids, such as blood (serum or plasma), urine, seminal plasma, and others, are closely associated with the physiological and pathological conditions of the human body [38–40]. Consequently, the detection of circulating nucleic acid has significant value in disease diagnosis and prognosis prediction, offering a minimally invasive and user-friendly approach to detecting relevant biomarkers. To further our understanding, we intend to investigate and elucidate the expression level of *LINC00467* in the TGCT patient serum and other body fluids, as well as its clinical significance. Our aim is to establish a novel, minimally invasive, and user-friendly diagnostic, classification, and prognosis prediction method based on *LINC00467* expression levels.

## Conclusion

We found that the silencing of *LINC00467* plays an important role in TGCT by inhibiting cell proliferation through the regulation of proteins, such as p21 and PCNA. The testosterone-stimulated expression of *LINC00467* may promote the development of TGCT. This study also provides a theoretical and experimental basis for drug research that targets *LINC00467* for the treatment of TGCTs.

## Supplemental Data
